# The Development and Exploratory Psychometric Properties of the Traumatic and Routine Stressors Scale on Emergency Nurses (TRSS-EN)

**DOI:** 10.3390/ijerph17061963

**Published:** 2020-03-17

**Authors:** Manuel Campillo-Cruz, José Luís González-Gutiérrez, Juan Ardoy-Cuadros, Juan José Fernández-Muñoz

**Affiliations:** Department of Medicine and Surgery, Psychology, Preventive Medicine and Public Health and Immunology, Medical Microbiology and Nursing and Stomatology, Faculty of Health Sciences, University Rey Juan Carlos, Avda. Atenas s/n, 28922 Alcorcón, Madrid, Spain; joseluis.gonzalez@urjc.es (J.L.G.-G.); juan.ardoy@urjc.es (J.A.-C.); juanjose.fernandez@urjc.es (J.J.F.-M.)

**Keywords:** emergency nursing, mental health and illness, post-traumatic stress disorder, routine stressors, traumatic stressors

## Abstract

Emergency nurses are exposed to traumatic events and routine stressors, both of which can lead to the development of PTSD (Post Traumatic Stress Disorder) symptomatology. However, there are currently no instruments designed to assess the impact and frequency of such sources of stress in nurses. The Traumatic and Routine Stressors Scale on Emergency Nurses (TRSS-EN) was built for this purpose. A sample of 147 emergency nurses from three hospitals in Madrid (Spain) completed this 13-item scale. The analyses showed a factorial structure composed of two factors. The first is characterized by items regarding traumatic and stressful events and procedures of severe magnitude (traumatic stressors), and the second by items related to stressful events and procedures of moderate magnitude (routine stressors) but hypothesized to possess a substantial traumatic potential. Analyses provided evidence of both adequate internal consistency (Cronbach’s α = 0.92; first factor α = 0.91 and second factor α = 0.86) and test–retest reliability. In addition, concurrent validity also proved to be satisfactory. In short, TRSS-EN seems to be a reliable and valid tool in a healthcare emergency nursing setting for screening the frequency and impact of exposure to everyday work-related traumatic stressors, either event-related or routine.

## 1. Introduction

Nurses at emergency services are exposed to traumatic and stressful events of different types and severity as a part of their everyday work. It is well known that exposure to both severe traumatic events [1] and to less significant, routine events [2] may potentially produce general psychiatric and, in particular, PTSD (Post Traumatic Stress Disorder) symptomatology. However, effects of such events on the mental health of healthcare professionals have been insufficiently investigated, although some studies show that such exposure induces vulnerability to chronic and post-traumatic stress as well as an increase in depressive and somatic symptomatology [3]. To our knowledge, the scales currently available that are intended to assess the impact of stressors in an everyday working setting for emergency nurses do not differentiate between the exposure to traumatic events and routine stressors. Furthermore, these scales do not consider the frequency of the exposure to such events.

### Background

Exposure to stressful or traumatic events is related to the development of different specific disorders, as stated in the APA’s (American Psychiatric Association) DSM-5 [4] or in the recently approved WHO ICD-11 [5]. The new ICD-11 differentiates between stressful events within the normal range of life experiences and those of an extreme or horrific nature (potentially traumatic events). Both can trigger the development of a disorder, but not in all individuals, and different kinds of stressors may potentially lead to different disorders (i.e., adjustment disorders or post-traumatic stress disorder) [5]. Accordingly, some authors distinguish between “traumatic” and “routine” stressors, with traumatic events referring to shocking, scary, or dangerous experiences that can affect someone emotionally and physically [1] and routine stressors referring to other less significant, albeit more frequent sources of stress, which are still assumed to possess substantial potential for trauma [6,7].

Whereas some authors have found that, in the general population, there is a direct relationship between traumatic event severity on the one hand and PTSD symptomatology severity on the other [8,9], the evidence is not conclusive. In fact, several studies failed to find this relationship [10,11]; therefore, it is not clear that more severe traumatic events increase the probability of developing symptomatology or that this symptomatology is more severe.

In contrast, there is an extensive literature on the relationships between more frequent routine stressors and psychiatric symptomatology [12,13,14], but its capability to produce PTSD symptomatology is less well known [7,15]. Nevertheless, some studies have shown that common stressors such as marital problems, divorce or unemployment may produce PTSD-like symptomatology [16], and that other common stressors such as family problems, imprisonment or severe illness of relatives may produce higher rates of PTSD-like symptoms than traumatic events do [17].

Nursing staff working at emergency services must deal with both work-related traumatic events and routine stressors, and this seems to be related to diverse forms of symptomatology, including PTSD symptoms [2,18]. In spite of this, research on the differential association of both kinds of stressors to PTSD is still in its early stages. While some studies suggest a positive relationship between stressor severity and PTSD symptom severity [1,3,19], others do not and also highlight the potential risk resulting from the exposure to other more frequent and less significant routine stressors [11,20]. It could be expected that repeated exposure to especially intense or relevant stressors could lead to sensitization (psychological awareness) [21,22], accordingly increasing the harmful potential impact of such events. On the contrary, repeated exposure to less threatening, more routine and frequent events could lead to habituation. But habituation or sensitization not only depend on the frequency or intensity of stimuli, but on the significance or relevance for the organism. Therefore, the results showing the harmful potential of routine stressors could be explained by their psychological relevance, namely, their perceived threat. A repeated stimulus perceived as threatening could lead to sensitization (if not for everyone, for several individuals). Further, more intense, traumatic events, could be evaluated as less threatening by emergency nurses, as they are connatural to their profession [1,23], so that they could lead to habituation, rather than to sensitization. Nurses could often feel that many routine stressors (i.e., dealing with relatives of patients, delivering good quality of care, dealing with aggression) are threatening because they are not specifically prepared for dealing with them, the working procedures may be not as clear as they should be, as well as the expected outcomes; additionally, these events are frequent. Therefore, the perceived threat of an event is not just a question of intensity. Many variables could impact on the perceived stress generated for a concrete event for different people. Additionally, different coping strategies may be displayed for reducing the impact of such events. Hence, the study of routine stressors as significant risk factors for developing PTSD and other psychiatric symptomatology in nurses is a relevant question. Recent studies with other emergency public servant professionals, such as police officers [7] and firefighters [24], have shown that stressors of moderate to high intensity are more harmful than very intense ones, depending on factors such as frequency of presentation and differential use of coping strategies, among others.

Despite the risk that repeated exposure to traumatic events and to routine stressors at emergency services represents for healthcare staff in general, and nurses in particular, there are no standardized instruments aimed at estimating the emotional impact caused by the exposure to both sources of disturbance. Exposure to traumatic events has usually been studied in emergency nurses by non-standardized direct questions about frequency and perceived emotional impact [1,3,25], although no specific instruments exist which have gone through the process of psychometric validation. The measurement of routine stressors in nurses has thus been somewhat neglected, as existing instruments [26,27,28] are far from considering other daily sources of occupational stress which can lead to consequences beyond the already dramatic (but not so significant) results covered by the expression “psychological strain” [29]. As an exception, the recently developed “Stressor scale of Emergency Nurses” [30] provides coverage of sources of stress which can be conceptually considered major or routine traumatic stressors, although they are not functionally clustered by the instrument in this way. Consequently, examination of the validity of the instrument under the umbrella of such a bidimensional conceptual differentiation has not been empirically addressed by Yuwanich et al., [26]. So far, despite its unquestionable strengths, the Stressor scale of Emergency Nurses is not suitable to be used in the way that is argued along the present study [18].

For these reasons, it seemed timely to build a normative scale to reliably assess the frequency of exposure to both work-related traumatic events and routine stressors (less significant, albeit more frequent sources of stress, which are still hypothesized to possess substantial potential for trauma) as well as the impact of such events on emergency nurses. The purpose of this study was to build a psychometric instrument to assess the severity and the frequency of exposure to severe traumatic events usually faced by emergency nurses, and to routine stressors which are usual in an everyday work scenario and which may eventually increase the risk of developing traumatic symptomatology. This article presents the Traumatic and Routine Stressors Scale on Emergency Nursing (TRSS-EN) and analyzes its psychometric properties.

## 2. Materials and Methods

### 2.1. Ethical Approval

The study protocol was approved by the research ethics committee of the University Rey Juan Carlos (number 030320162116).

### 2.2. Design

The study was of a quantitative design, comprising a cross-sectional survey to test construct and concurrent validity, together with a longitudinal survey on a small subsample in order to examine the instrument’s test–retest reliability.

### 2.3. Participants and Procedure

The sample consisted of 147 emergency nurses (128 women and 19 men), working in three hospitals in Madrid (Spain) and aged between 24 and 61. The mean age of respondents was 40.41 (SD = 8.32), and they had been working in the same department for a mean of 9.9 years (SD = 6.69). In total, 111 spent more than 75% of their working time in direct contact with their patients (Table 1 presents a detailed description of the sample). Participants were required to have worked in the same department for at least one year. They were also required to be free of severe psychopathology (DSM-5) [4] or severe chronic physical illness. For each hospital, we applied a simple random sampling between the whole of nursing workers from the three hospitals. We assumed a confidence level of 95% and a level of heterogeneity of 50%.

A total of 218 nursing professionals were initially involved. Thirteen participants were excluded on application of inclusion criteria, and 68 did not return complete questionnaires (response rate = 71.7%). The study proceeded over 16 months, from February 2017 until June 2018, during which researchers were in contact with participants. Initially, brief meetings were held in which the research aims were presented and the inclusion criteria discussed. All participants who voluntarily agreed to participate provided signed informed consent and were instructed on how to complete the questionnaire items, which were administered via the Internet. Participants were given a month to return the instruments, during which a reminder was sent by email. Exactly three weeks after this deadline, a randomly selected subsample of 40 participants, 7 men and 33 women (mean of age = 40.51; SD = 8.19), was asked to fill in the questionnaire for a second time in order to examine the instrument’s test–retest reliability. In all instances, participant anonymity was preserved by means of the use of codes.

The study was conducted in accordance with the Declaration of Helsinki (World Medical Association 2013) [31]. Ethics approval was granted by the Ethics Committee of the University Review Board.

### 2.4. Instruments

#### 2.4.1. The Spanish Version of the Symptom Assessment-45 Questionnaire (SA-45)

Psychological distress and somatic complaints were measured by means of the validated Spanish version [32] of the Symptom Assessment-45 Questionnaire (SA-45) [33]. The SA-45 has been found to be a good and shorter alternative to the SCL-90-R [34]. This instrument is a 45-item measure that assesses the presence of specific symptoms over the past seven days. The SA-45 consists of nine 5-item subscales: “Depression”, “hostility”, “interpersonal sensitivity”, “somatization”, “anxiety”, “psychoticism”, “obsessive-compulsive”, phobic anxiety” and “paranoid ideation”. All items are rated on a 5-point Likert scale from “not at all” to “extremely”. Coefficient Alpha = 0.95. Cronbach’s alpha for each of the questionnaire scales, the majority being equal to or greater than 0.80. Appropriate convergent and discriminant validity.

#### 2.4.2. The Posttraumatic Diagnostic Scale for DMS-5 (PDS-5)

The Posttraumatic Diagnostic Scale for DMS-5 (PDS-5) [35] is a 24-item measure which we used to assess PTSD symptom severity in the previous month according to DSM-5 criteria [4]. The PDS-5 consists of four subscales: “Intrusion” (5 items), “avoidance” (2 items), “changes in mood and cognition” (7 Items) and “arousal and hyperreactivity” (6 Items). All these items are rated on a 5-point Likert scale from “not at all” to “6 or more times a week/severe”. Four additional items ask about distress and interference caused, as well as the onset and duration of symptoms. A high score on the SA-45 represents a high level of psychiatric symptomatology. Cronbach’s α = 0.92, test–retest reliability (r = 0.90). Good convergent validity.

#### 2.4.3. Traumatic and Routine Stressors Scale on Emergency Nurses (TRSS-EN)

The Traumatic and Routine Stressors Scale on Emergency Nurses (TRSS-EN) was developed from the list of traumatic stressors (10 items) empirically isolated in emergency nurses [1], plus three items included ad hoc based on the previous literature review about the most common traumatic events that health personnel are exposed to [36]. The objective was to identify, together with some of the most characteristic traumatic events of the nursing profession, several routine stressors capable of producing PTSD symptomatology, whose coexistence with traumatic events has been systematically suggested by previous research [17]. To do this, it was initially hypothesized that both routine stressors and traumatic events constitute a continuum, with the former somewhat lower on the scale in terms of its ability to emotionally impact, but also higher than traumatic events in terms of its frequency of presentation. Once applied, factorial analysis should work on this continuum by grouping the most emotionally impacting stressors (traumatic events) and differentiating them from the other stressors of somewhat lower emotional impact (routine stressors). The resulting tool was a 13-item questionnaire representing traumatic and routine stressors, on which nurses indicated one by one the frequency with which they were exposed to the stressful event during the previous six months (frequency of exposure) on a 7-point Likert scale, where 1 is “fewer than three times in six months” and 7 is “every day” (Frequency scale), and the degree to which they were emotionally affected by them (emotional impact of the stressful event), using a second 7-point Likert scale, where 1 is “without emotional impact” and 7 is “maximum emotional impact” (Impact scale). The final scale provides six indexes: “Emotional Impact of traumatic stressors”, “Emotional Impact of routine stressors”, “Frequency of traumatic stressors”, “Frequency of routine stressors”, “Total Impact of traumatic stressors”, and “Total Impact of routine stressors”, as described later.

### 2.5. Analysis

#### Psychometric Analysis of TRSS-EN

SPSS 25.0 (IBM, Armonk, NY, USA) was used to apply the exploratory factor analysis, reliability analysis and correlations. Assumptions such as multivariate normality were tested (Kolmogorov test *p* > 0.05). The number of factors was identified with several criteria, for instance, Scree Plot and eigenvalue greater than 1 [37]. The estimation method was maximum likelihood and the rotation method was varimax. The internal consistency of the scale was checked through Cronbach Alpha and an item homogeneity index.

In order to shed additional light on the construct validity of the instrument, and under the hypothesis that the emotional impact of traumatic events would be higher than that caused by routine stressors, a factorial analysis was followed by the examination of the differences (Student’s t-test for paired samples) between scores on the paired indexes of the instrument as a function of exposure to the presumed differential magnitude of the event: “Emotional Impact of traumatic stressors” versus “Emotional Impact of routine stressors”; “Frequency of traumatic stressors” versus “Frequency of routine stressors”; and “Total Impact of traumatic stressors”, versus “Total Impact of routine stressors”.

Concurrent validity was examined by correlating the six indexes that the TRSS-EN provides with Psychological distress and somatic complaints (general symptomatology assessed by SA-45 dimensions) and with post-traumatic stress reactions (post-traumatic symptomatology assessed by PDS-5 dimensions). Steiger’s z were calculated in order to test differences between the correlations of traumatic versus routine stressors, with each corresponding SA-45 and PDS-5 dimension.

Finally, test–retest reliability (with an interval of three weeks) was assessed at the individual item level (at the impact and frequency levels of measurement) and also at the global level of the six general indexes. At both levels, agreement was analyzed with a two-way random effects single measure intraclass correlation coefficient (ICC 2.1); ICCs were classified as follows: “excellent” (≥0.81), “good” (0.61–0.80), “moderate” (0.41–0.60), “poor” (≤0.40) [38,39,40].

## 3. Results

### 3.1. Descriptive Analysis and Internal Consistency

The descriptive results of the TRSS-EN are shown in Table 2. The Kolmogorov test for all items was *p* > 0.05. Means, standard deviation, skewness and kurtosis indices, homogeneity index and α, if item is deleted, are included for each item of the TRSS-EN. The lowest value was 3.26 (SD = 1.43) for the item “Dealing with aggression, violence and threat” and the highest score was 5.09 (SD = 2.00) for the item “Dealing with relatives of victims/patients”. Cronbach’s alpha for the TRSS-EN scale was 0.92. With the split sample method, the alpha for the first half was 0.88 and 0.82 for the second. The correlation between both halves was r = 0.84, *p* < 0.01, the Spearman coefficient was 0.91 and Guttman’s coefficient was 0.90.

### 3.2. Exploratory Factor Analysis

**Figure 1:**
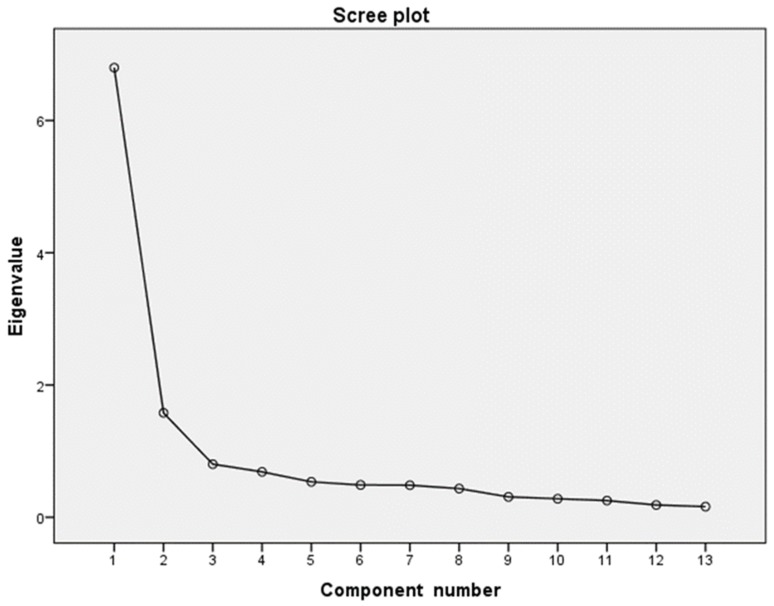
Scree plot of eigenvalues for the TRSS-EN Items, *N* = 147 for the impact scale in TRSS-EN. The scree plot displayed in Figure 1 depicts a sharp descent in the curve, or point of inflection, at the second component. [37] recommendation of retaining only components to the left of the inflection point supports a single-component solution.

The scree plot display shows a factorial solution with two factors (Figure 1) for the impact scale in TRSS-EN. The eigenvalues for these factors were >1: 6.79 for the first and 1.57 for the second factor. The communality values ranged from 4.64 to 7.59. According to these criteria, the factorial solution was composed of two factors (Table 3). The first comprised the following items: 1 “Dealing with sudden death of young persons”, 2 “Dealing with death or resuscitation of a baby or young child”, 3 “Handling victims of car and train crashes”, 4 “Confrontation with physical trauma and burns patients”, 5 “Dealing with suicide”, 10 “Confrontation with child abuse and negligence” and 11 “Exposure to sudden death”, with factorial weights between 0.682 and 0.846. This factor, labeled “Traumatic stressors”, was made up of traumatic events and stressors of great magnitude. Cronbach’s alpha for this factor was 0.911 and Omega index was 0.857. The second factor was composed of items 6 “Dealing with aggression, violence and threat”, 7 “Inability to deliver good quality of care”, 8 “Inability to help chronically ill patients”, 9 “Dealing with relatives of victims/patients, 12 “Dealing with psychiatric patients”, and 13 “Management of dead bodies”, with factorial weights between 0.562 and 0.819. This second factor was called “Routine stressors” and comprised events and stressors of moderate magnitude. Cronbach’s alpha for this factor was 0.862 and Omega index was 0.833. Several items showed factorial weights in both dimensions. The percentage of total variance explained was 64.44%, 35.63% for the first factor and 28.81% for the second.

The two dimensions of the factorial solution were similar to those found in previous studies [6,7], focusing on samples of police officers who were exposed to different types of stressful events compared to the sample of this study.

### 3.3. Validity Assessments

According to the result of the factor analysis, the two factors were interpreted as subscales and, in order to obtain a simple measure of each subscale, the summation of the ratings were calculated for each subject, giving rise to the indexes named “Emotional Impact of traumatic stressors” (resulting from the summation of the scores of perceived emotional impact in items 1, 2, 3, 4, 5, 10 and 11) (M = 4.56, SD = 1.44, 95% CI: 1.00–7.00) and “Emotional Impact of routine stressors” (resulting from the summation of the scores of perceived emotional impact in items 6, 7, 8, 9, 12 and 13) (M = 3.84, SD = 1.44, 95% CI: 1.00–6.67). Next, frequency ratings for each stressful event were grouped following the identical factorial structure, giving rise to the indexes of “Frequency of traumatic stressors” (resulting from the summation of the frequency with which items 1, 2, 3, 4, 5, 10 and 11 were experienced) (M = 1.98, SD = 0.84, 95% CI: 1.00–5.14) and “Frequency of routine stressors” (resulting from the summation of the frequency with which items 6, 7, 8, 9, 12 and 13 were experienced) (M = 3.76, SD = 1.34, 95% CI: 1.00–7.00). Finally, the individual product, item by item, of emotional impact by frequency of exposure, gives rise to two new indexes: “Total Impact of traumatic stressors” (resulting from the summation of the products of emotional impact and frequency of items 1, 2, 3, 4, 5, 10 and 11) (M = 427.09, SD = 240.76, 95% CI: 5.00–1764.00) and “Total Impact of routine stressors” (resulting from the summation of the products of emotional impact and frequency of items 6, 7, 8, 9, 12 and 13) (M = 532.57, SD = 300.71, 95% CI: 36.00–1560.00).

Once the six indexes were created, analyses of differences between the paired scores showed that scores for “Emotional Impact of traumatic stressors” were higher than those for “Emotional Impact of routine stressors” (t = 7.70; *p* = 0.01). Conversely, scores for “Frequency of routine stressors” were higher than those for “Frequency of traumatic stressors” (t = −18.73; *p* = 0.01), and scores for “Total Impact of routine stressors” were also higher than scores for “Total Impact of traumatic stressors” (t = −5.45; *p* = 0.01).

Table 4 represents zero order correlations between TRSS-EN global indexes, and general and PTSD symptomatology (SA-45 and PDS-5 dimensions, respectively). The overall impression is that emotional impact of routine stressors, but not emotional impact of traumatic stressors, correlated with general and specific PTSD symptomatology. However, in terms of the correlations between the total impact (which considers the frequency of exposure) of both traumatic and routine stressors with symptomatology, only some differences were observed for general symptomatology. As additional information, no significant correlations were observed between years of experience and general symptoms or PTSD, nor among nurses having children and exposure to death or childhood abuse (all above 0.05).

### 3.4. Test–retest

No differences were observed between the test–retest subsample (*n* = 40) and the whole sample (*n* = 147) in terms of age (t = 0.07; *p* = 0.47) or sex distribution (chi square = 0.15; *p* = 0.70). For the global indexes, test–retest reliability was good, with ICC-values between 0.66 and 0.70 (Emotional Impact of traumatic stressors = 0.73; Emotional Impact of routine stressors = 0.66, Frequency of traumatic stressors = 0.74, Frequency of routine stressors = 0.68, Total Impact of traumatic stressors = 0.75, and Total Impact of routine stressors = 0.70).

At the individual item level (see Table 5), test–retest reliability was good in seven items (53%) both at the impact and frequency levels of measurement, whereas it was moderate in six items (47%) at the impact level of measurement, and in five items (38.46%) at the frequency level of measurement. Only one item (7.69%) at the frequency level of measurement (“sudden death”) yielded a lower ICC-value.

## 4. Discussion

To date, no standardized instruments have been created which allowed the joint estimation of the frequency and emotional impact of traumatic events and routine stressors in the emergency nursing profession. Only rare attempts have been made that did not consider the necessity of studying the stressful potential of both factors and their link with diverse forms of symptomatology, including post-traumatic stress [1,11,20]. However, a need to address this issue has become evident, since it seems unclear that traumatic events of great magnitude are inevitably more related to the development of post-traumatic symptomatology than other less significant, moderate routine stressors [2,17].

To our knowledge, TRSS-EN is the first tool developed to consider the existence of two distinguishable levels of severity in the everyday experiences of emergency nurses. It allows the simultaneous assessment of both the frequency of exposure and the perceived emotional impact of these factors. TRSS-EN is a 13-item questionnaire showing satisfactory validity, and with internal consistency and test–retest reliability which was found to be excellent in the present sample.

The analyses showed firstly that the factorial solution was composed of two dimensions, these two dimensions corresponding to, respectively, traumatic events and procedures of significant magnitude (traumatic stressors) and stressful events and procedures of moderate magnitude (routine stressors). This solution fits well with current proposals concerning the existence of a cluster of highly stressful factors with highly “traumatic” potential [41,42], distinguishable from a constellation of routine, nontraumatic stressors with even greater potential to generate general and post-traumatic symptomatology [7,17]. Thus, in the present study, correlations of both factors and their resulting indexes with general (SA-45) [32] and specific PTSD symptomatology (PSD-5) [35] pointed again in this direction by yielding a stronger association of routine stressors with both forms of symptomatology. These results represent significant evidence in favor of both the construct and concurrent validity of the instrument. Secondly, due to the correspondence between TRSS-EN items and the set of traumatic events identified [1], as especially relevant in the emergency nursing profession, the content validity of the instrument also seems to be confirmed. Finally, the instrument’s reliability, estimated by internal consistency, split sample and split-half methods, was shown to be highly satisfactory. Similarly, the test–retest also showed good reliability, even considering the context of systematic change inherent in this type of construct.

### Limitations

Some limitations of the present study should be mentioned. First, in spite of the adequate reliability and validity of this instrument, it appears necessary to analyze the factorial structure and the degree of homogeneity of the proposed items in different samples of nurses. Such studies would confirm the dimensions obtained and their relationships with other psychological constructs. Similarly, this would help provide a better assessment of the events experienced by emergency nurses and lead to better interventions involving strategies to improve the impact of these events. Second, considering the special characteristics of this population (emergency nursing staff), repeatedly exposed to traumatic events of differing emotional magnitudes in their work setting on a daily basis, it is possible that PDS-5 [35] cannot seamlessly cover the complete spectrum of post-traumatic implications characteristic of the nursing profession. In the absence of a specific questionnaire with the ability to register PTSD symptoms in this particular group of professionals, as well as for the limited correlations observed between the dimensions of TRSS-EN and PTSD symptomatology, it could be interesting to further explore this question in future research. Third, given the cross-sectional nature of the study, it is important to be cautious in interpreting the sense of the relationships found, examining not only the concurrent presence of symptomatology, but also the ability of TRSS-EN to predict its future occurrence. Longitudinal analysis to examine this specific question also seems necessary. Fourth, the “Stressor scale of Emergency Nurses” [30] provides a coverage of sources of stress, some of them capable of being conceptually comparable to “traumatic stressors” and routine stressors”, respectively. Since this scale had not yet been published at the onset of the present study, convergence with TRSS-EN was not examined, something which should be studied in the future. Finally, although this study constitutes a good representation of the real distribution of women and men in the professional nursing setting, results should preferably be generalized to women. Future studies should expand the examination of the psychometric proprieties of TRSS-EN in the male population.

## 5. Conclusions

The present study provides preliminary evidence in a sample of emergency nurses to support the hypothesis that TRSS-EN seems to be a reliable and valid tool for exploring the traumatic circumstances in which the work of emergency nurses is performed. TRSS-EN is the first tool which allows the simultaneous assessment of the frequency of exposure to and the perceived emotional impact of stressful events and procedures of the emergency nursing profession at two levels of severity (infrequent/traumatic and routine/moderate). Both are capable of causing diverse symptomatology, including post-traumatic stress. These characteristics make it a promising tool for the study of stress exposure levels of emergency nurses in order to plan appropriate interventions at individual and organizational levels.

## Figures and Tables

**Table 1:** Basic descriptors of study participants as a function of their demographic and professional characteristics.

Descriptive Data	Mean	SD	*n*	Percentage
Age (*N* = 144)	40.41	8.32		
Children (*N* = 147)	0.99	1.02		
Years of experience in the profession (*N* = 147)	16.48	7.33		
Years of experience in the same job (*N* = 147)	9.9	6.69		
**Characteristics**				
Gender			147	100
Male			19	12.9
Female			128	87.1
Marital status			145	100
With regular partner			115	79.3
Single			30	20.7
Studies			146	100
DUE			88	60.3
General Nurse			11	7,5
TCAE			43	29.5
Others			4	2,7
Employment situation			147	100
Permanent			72	49
Non-permanent			75	51
Percentage with patients			146	100
Less than 25%			8	5.5
From 25% to 50%			2	1.4
From 50% to 75%			25	17.1
More than 75%			111	76

Note: Children refers to the number of children of the participants. DUE (“Diplomado Universitario de Enfermería”) is equivalent to General Nurse; TCAE (“Técnico en Cuidados Auxiliares de Enfermería”) is equivalent to nursing assistant.

**Table 2:** Means, standard deviations, skewness, kurtosis, item homogeneity and α if item deleted for the impact scale in TRSS-EN.

Item	Statement	Mean	SD	S	K	IH	Alpha
TRSS-EN_1	Dealing with sudden death of young persons	4.27	1.67	−0.20	−0.75	0.39	0.78
TRSS-EN_2	Dealing with death or resuscitation of a baby or young child	4.68	1.62	−0.45	−0.50	0.41	0.78
TRSS-EN_3	Handling victims of car and train crashes	4.34	1.55	−0.35	−0.48	0.57	0.76
TRSS-EN_4	Confrontation with physical trauma and burns patients	4.06	1.74	−0.35	−0.83	0.49	0.77
TRSS-EN_5	Dealing with suicide	3.86	1.60	−0.01	−0.51	0.44	0.78
TRSS-EN_6	Dealing with aggression, violence and threat	3.26	1.43	0.18	−0.45	0.57	0.77
TRSS-EN_7	Inability to deliver good quality of care	3.55	1.62	0.04	−0.66	0.41	0.78
TRSS-EN_8	Inability to help chronically ill patients	3.90	1.57	−0.02	−0.59	0.50	0.77
TRSS-EN_9	Dealing with relatives of victims/patients	5.09	2.00	−0.89	−0.42	0.24	0.79
TRSS-EN_10	Confrontation with child abuse and negligence	4.00	1.46	0.14	−0.20	0.52	0.77
TRSS-EN_11	Exposure to sudden death	5.09	2.15	−0.87	−0.66	0.11	0.80
TRSS-EN_12	Dealing with psychiatric patients	4.97	1.92	−0.75	−0.57	0.40	0.79
TRSS-EN_13	Management of dead bodies	3.82	1.62	0.19	−0.77	0.43	0.78

*N* = 145, standard error of skewness = 0.201; standard error of kurtosis = 0.400.

**Table 3:** Matrix of rotated components and factorial weighs of the items for the impact scale in TRSS-EN.

		Components		Communalities
Item	Statement	TS	RS	
TRSS-EN_1	Dealing with sudden death of young persons	0.704	0.325	0.602
TRSS-EN_2	Dealing with death or resuscitation of a baby or young child	0.846	0.101	0.726
TRSS-EN_3	Handling victims of car and train crashes	0.686	0.372	0.609
TRSS-EN_4	Confrontation with physical trauma and burns patients	0.682	0.455	0.673
TRSS-EN_5	Dealing with suicide	0.725	0.300	0.617
TRSS-EN_6	Dealing with aggression, violence and threat	0.559	0.562	0.628
TRSS-EN_7	Inability to deliver good quality of care	0.212	0.819	0.715
TRSS-EN_8	Inability to help chronically ill patients	0.169	0.803	0.673
TRSS-EN_9	Dealing with relatives of victims/patients	0.270	0.767	0.661
TRSS-EN_10	Confrontation with child abuse and negligence	0.828	0.144	0.707
TRSS-EN_11	Exposure to sudden death	0.835	0.249	0.759
TRSS-EN_12	Dealing with psychiatric patients	0.196	0.712	0.546
TRSS-EN_13	Management of dead bodies	0.283	0.620	0.464
	% explained variance	35.63	28.81	
	Cronbach alpha Omega	0.911 0.857	0.862 0.833	

Note: TS = Traumatic Stressors (Traumatic events and Great magnitude Stressors); RS = Routine Stressors (Events and Stressors of Moderate magnitude). The factor weights in item 6, although similar for the first component, were slightly higher in the second component. This item is more related to RS. It is not perceived as a traumatic event and it does not imply an imminent risk of death.

**Table 4:** Bivariate correlations between the six global indexes of TRSS-EN, general symptoms (SA-45) and symptoms of PTSD (PDS-5).

			Emotional Impact			Frequency			Total Impact		
	Mean	SD	TS	RS	Steiger’s Z	TS	RS	Steiger’s Z	TS	RS	Steiger’s Z
Psychopathological symptoms (SA-45)	31.29	24.34	0.14	0.30 **	−2.44 *	0.17 *	0.22 **	−0.61	0.21 *	0.35 **	−2.05 *
Hostility	0.47	0.62	0.16	0.21 **	−0.78	0.14	0.16	−0.17	0.23 **	0.26 **	−0.52
Somatization	1.09	0.87	0.12	0.23 **	−1.59	0.05	0.12	−0.86	0.05	0.21 *	−2.23 *
Depression	0.84	0.75	0.04	0.24 **	−2.82 **	0.14	0.22 **	−1.82	0.13	0.31 **	−2.50 *
Obsessive−compulsive	0.93	0.77	0.20 *	0.31 **	−1.68	0.11	0.15	−0.49	0.21 *	0.30 **	−1.33
Anxiety	0.86	0.70	0.19 *	0.30 **	−1.59	0.18 *	0.22 **	−0.39	0.26 **	0.33 **	−1.01
Interpersonal sensitivity	0.82	0.79	0.08	0.26 **	−2.53 *	0.20 *	0.23 **	−0.31	0.19 *	0.32 **	−1.88
Phobic anxiety	0.25	0.46	0.00	0.13	−1.76	0.06	0.04	0.26	0.08	0.13	−0.80
Paranoid ideation	0.75	0.65	0.04	0.24 **	−2.79 **	0.21 **	0.22 **	−0.04	0.15	0.29 **	−2.05 *
Psychoticism	0.25	0.42	0.13	0.25 **	−1.78	0.17 *	0.27 **	−1.17	0.25 **	0.37 **	−1.70
Symptoms of PTSD (PDS−5)	12.77	13.40	0.13	0.31 **	−2.55 *	0.08	0.10	−0.17	0.14	0.19	0.43
Intrusion	3.58	3.68	0.08	0.27 **	−2.74 **	0.12	0.07	0.81	0.18	0.16	0.30
Avoidance	1.52	1.68	0.19	0.31 **	−1.87	0.05	0.10	−0.60	0.13	0.18	−0.73
Changes in mood and cognition	3.62	4.76	0.04	0.23 *	−2.78 **	0.07	0.13	−0.69	0.06	0.17	−1.58
Arousal and hyperreactivity	4.04	4.78	0.20*	0.31 **	−1.54	0.05	0.07	−0.18	0.14	0.19	−0.59

** *p* < 0.01; * *p* < 0.05. Note: TS = Traumatic Stressors (Traumatic events and Great magnitude Stressors); RS = Routine Stressors (Events and Stressors of Moderate magnitude).

**Table 5:** Test–retest reliability as indicated by item-to-item interclass correlation coefficients.

	Impact						Frequency					
	Test		Retest				Test		Retest			
	Mean	SD	Mean	SD	t	CCI	Mean	SD	Mean	SD	t	CCI
1	4.80	1.60	4.95	1.64	−0.67	0.63	1.72	0.96	1.92	0.97	−1.27	0.47
2	4.48	2.25	5.40	2.06	−2.40 *	0.75	1.19	0.62	1.08	0.36	1.67	0.69
3	3.80	1.57	3.97	1.51	−0.75	0.55	3.35	2.20	3.15	1.95	0.82	0.73
4	3.77	1.64	3.95	1.58	−0.76	0.60	2.52	1.66	2.75	1.53	−1.00	0.60
5	4.20	1.87	4.54	1.73	−1.27	0.58	2.23	1.51	2.41	1.55	−0.89	0.67
6	4.55	1.39	4.70	1.71	−0.61	0.50	2.95	1.60	3.10	1.69	−0.59	0.53
7	5.00	1.63	4.67	1.65	1.24	0.49	4.10	2.23	4.40	2.05	−1.06	0.65
8	4.08	1.78	3.97	1.68	0.45	0.66	3.27	2.01	3.75	1.75	−1.93	0.65
9	4.18	1.62	4.15	1.60	0.09	0.45	4.51	2.27	4.95	−1.27	−1.27	0.51
10	5.27	2,14	5.62	1.78	−1.38	0.69	1.49	1.24	1.40	0.36	0.36	0.42
11	4.76	2.00	5.19	1.81	−1.65	0.64	1.51	0.87	1.57	−0.29	−0.29	0.39
12	4.05	1.60	4.00	1.59	0.24	0.65	4.74	2.14	4.74	1.92	0.00	0.67
13	3.33	1.75	3.33	1.34	0.00	0.67	2.68	1.71	2.69	1.38	−0.11	0.57

* *p* < 0.05.
